# Comparative Outcomes of Radical Surgery, Conservative Surgery, and Percutaneous Treatment for Active Hepatic Cystic Echinococcosis: A Retrospective Cohort Study

**DOI:** 10.7759/cureus.118952

**Published:** 2026-11-01

**Authors:** Rajesh K Nanjundaiah, Vinod B N Kumar, Vikram Ramamurthy, Naveen Narayan, Pallavi H R

**Affiliations:** 1 General Surgery, Adichunchanagiri Institute of Medical Sciences, Nagamangala, IND; 2 General Surgery, Dr. B.R. Ambedkar Medical College, Bengaluru, IND; 3 Plastic Reconstructive and Aesthetic Surgery, Adichunchanagiri Institute of Medical Sciences, Nagamangala, IND

**Keywords:** conservative surgery, hepatic cystic echinococcosis, hydatid cyst, pair, percutaneous treatment, radical surgery, retrospective cohort study, who-iwge classification

## Abstract

Background: Treatment of active hepatic cystic echinococcosis (CE) depends on cyst stage and clinical features. Comparative outcomes of radical surgery, conservative surgery, and percutaneous treatment remain uncertain. We compared outcomes across these strategies in a single-center cohort.

Methods: We retrospectively reviewed adults treated at a tertiary-care center in Karnataka, India, between January 2020 and December 2024. Patients were grouped by definitive intervention: radical surgery (n = 15), conservative surgery (n = 18), or percutaneous puncture, aspiration, injection, and re-aspiration (PAIR) (n = 8). Outcomes included treatment success, complications, hospital stay, and recurrence.

Results: World Health Organization Informal Working Group on Echinococcosis (WHO-IWGE) stage distribution differed among groups (p < 0.001): CE3b (daughter cysts within a solid matrix) accounted for 80.0% of the radical group, 55.6% of the conservative group, and 25.0% of the PAIR group. Treatment success was observed in 15/15 patients (100.0%) after radical surgery, 15/18 (83.3%) after conservative surgery, and 4/8 (50.0%) after PAIR (p = 0.007). Complications occurred in 2/15 (13.3%), 6/18 (33.3%), and 5/8 (62.5%), respectively (p = 0.038). Mean hospital stay was 6.1, 9.8, and 3.5 days, respectively (p < 0.001). Recurrence occurred in 0/15, 2/18 (11.1%), and 2/8 (25.0%).

Conclusion: Radical surgery had the highest observed treatment success and no recorded recurrence in this cohort. Because treatment groups differed by cyst stage and treatment was not randomized, these findings are exploratory and do not establish comparative superiority.

## Introduction

Cystic echinococcosis (CE) is a zoonotic parasitic disease caused mainly by the larval stage of *Echinococcus granulosus*. The liver is the most commonly affected organ. Hepatic cysts can remain asymptomatic for years, but enlargement or complications such as rupture, infection, or biliary communication can lead to substantial morbidity [1,2].

The World Health Organization Informal Working Group on Echinococcosis (WHO-IWGE) classifies cysts according to their ultrasound appearance and biological activity. CE1 and CE2 are active stages; CE3a and CE3b are transitional stages; and CE4 and CE5 are inactive stages. CE3b cysts contain daughter vesicles within a predominantly solid matrix, a feature that can complicate evacuation and treatment [3]. Staging helps guide management, but treatment decisions also depend on cyst size and location, symptoms, complications, biliary communication, patient factors, and available clinical expertise [4,5].

Management options include antiparasitic medication, percutaneous procedures, surgery, and imaging follow-up for selected inactive cysts. These approaches are not interchangeable across cyst stages. Percutaneous puncture, aspiration, injection, and re-aspiration (PAIR) is a minimally invasive, image-guided technique for hepatic CE. Under ultrasound guidance, the cyst is punctured percutaneously, and most of its fluid is aspirated. A scolicidal agent, such as hypertonic saline or ethanol, is then instilled and left in situ for a defined exposure time, after which the cavity is re-aspirated. Unlike percutaneous catheter drainage, PAIR does not usually leave a catheter in place. It is performed under albendazole cover to reduce the risk of secondary spread and is generally considered for selected cysts, with biliary communication a recognized contraindication because of the risk of scolicidal-induced biliary injury [4,5]. Cysts with daughter vesicles in a solid matrix (CE3b) are difficult to evacuate by simple aspiration, which is why PAIR is less established for these stages [3,4]. Therefore, conclusions about a treatment’s suitability should account for the characteristics of the patients and cysts receiving it.

Surgery ranges from conservative procedures, such as cyst unroofing and partial pericystectomy with management of the residual cavity, to radical procedures, such as total pericystectomy or anatomical liver resection. Radical procedures aim to remove the cyst and surrounding pericyst, while conservative procedures preserve more liver parenchyma but leave a residual cavity requiring management. Each approach has potential benefits and risks, and the choice is influenced by cyst anatomy, proximity to vascular and biliary structures, and surgical expertise [6]. Comparative studies of radical and conservative surgery have reported different outcomes, while reviews have noted limitations in the available evidence and variation in patient selection and study methods [7,8].

Evidence comparing surgical and percutaneous strategies across active hepatic CE stages remains limited, particularly in routine single-center practice. The aim of this study was to compare treatment success, periprocedural complications (including biliary leak or fistula), hospital length of stay, and cyst recurrence among adults with active or transitional hepatic CE managed with radical surgery, conservative surgery, or PAIR at a single tertiary-care center between January 2020 and December 2024. Because treatment was not randomly assigned, this study evaluates observed associations between treatment strategy and outcomes; it cannot establish that one strategy caused better outcomes.

## Materials and methods

Study design, setting, and ethical approval

We retrospectively reviewed consecutive adults who received definitive treatment for hepatic CE at Adichunchanagiri Institute of Medical Sciences, Nagamangala, India, between January 2020 and December 2024. Patients were eligible for the primary 12-month outcome if at least 12 months of post-treatment clinical and radiological follow-up were documented. Follow-up records were reviewed through June 2026. Four patients were excluded because they had less than 12 months of documented follow-up. Clinical records, radiology archives, operative reports, procedure records, and follow-up documentation were reviewed. The Institutional Ethics Committee (IEC) of Adichunchangiri Institute of Medical Sciences approved the study (IEC No. AIMS/IEC/316/2026). The study was designed, conducted, and reported in accordance with the Strengthening the Reporting of Observational Studies in Epidemiology (STROBE) Statement.

Patient selection and cohort definition

Patients were eligible if they met the following inclusion criteria: age ≥18 years; imaging-confirmed active or transitional hepatic CE classified as WHO-IWGE stage CE1, CE2, or CE3b (no patients with CE3a cysts were identified during the study period); primary definitive treatment performed at the study center; and a minimum of 12 months of documented post-procedure clinical and radiological follow-up. 

Patients were excluded based on the following exclusion criteria: inactive WHO-IWGE CE4 or CE5 cysts managed conservatively without intervention; patients treated previously for recurrent disease at another facility without baseline operative records; secondary or extrahepatic hydatid disease without primary hepatic involvement; incomplete clinical records or loss to follow-up prior to 12 months. 

During the study period, 52 patients were screened for eligibility. Eleven patients were excluded (four with inactive CE4/CE5 cysts, three with prior extra-institutional interventions lacking records, and four with less than 12 months of follow-up). The final cohort comprised 41 patients: 15 treated with radical surgery, 18 with conservative surgery, and eight with percutaneous PAIR. 

Baseline assessment and disease classification

Demographic, clinical, laboratory, and imaging variables were extracted from the medical record. This included age, sex, presenting symptoms, cyst diameter, number and location of cysts, WHO-IWGE stage, bilirubin level, and hydatid serology where available. The cyst stage was recorded from preprocedural imaging reports and classified as CE1, CE2, or CE3b. 

Treatment strategies

All patients received perioperative albendazole at 10-15 mg/kg/day two weeks prior to planned surgical or percutaneous intervention and continued for four weeks post-procedure for surgery and eight weeks post-procedure for PAIR, with biweekly liver function monitoring. PAIR was performed under ultrasound guidance using an 18-gauge needle; 20% hypertonic saline was instilled with an exposure time of 15-20 minutes prior to complete re-aspiration. Three patients in the PAIR group underwent conversion to open surgery due to biliary communication/thick cyst contents; per protocol, converted patients remained in the PAIR group under an intention-to-treat approach and were categorized as treatment failures.

Radical surgery comprised total pericystectomy or anatomical liver resection, whereas conservative surgery comprised cyst unroofing or partial pericystectomy with management of the residual cavity. Surgery was performed laparoscopically in some patients. Treatment was not randomly assigned. It was selected in routine clinical practice, and factors that may have influenced the choice included cyst stage, size and location, biliary communication, complications, and proximity to major vascular structures. Cyst stage (CE1, CE2, or CE3b) was recorded from the original preprocedural imaging reports. For PAIR, the cyst was punctured under ultrasound guidance with an 18-gauge needle, 20% hypertonic saline was instilled for 15-20 minutes, and the cyst was then completely re-aspirated. Patients converted to open surgery because of biliary communication or thick cyst contents (n = 3) remained in the PAIR group under an intention-to-treat approach and were classified as treatment failures.

Outcomes and follow-up

The primary outcome was treatment success, defined as complete cyst collapse/obliteration without recurrence or a secondary procedure. Secondary outcomes were treatment failure, conversion to surgery, biliary leak or fistula, other periprocedural complications, procedure duration, estimated blood loss, transfusion, hospital length of stay, and cyst recurrence. Complications were graded using the Clavien-Dindo classification. Patients underwent clinical review and ultrasound at one, three, six, and 12 months, then annually, with CT or MRI when clinically indicated. Median follow-up was 36 months.

Statistical analysis

Analyses were performed using IBM SPSS Statistics for Windows, Version 28 (Released 2021; IBM Corp., Armonk, New York, United States). Continuous variables were summarized as mean and standard deviation (SD) or median and interquartile range (IQR), as appropriate, and compared across groups using one-way analysis of variance or the Kruskal-Wallis test. Categorical variables were summarized as counts and percentages and compared using the chi-square test or Pearson’s chi-square test or Fisher-Freeman-Halton exact test when expected cell counts were small. Recurrence-free survival was estimated using the Kaplan-Meier method and compared using the log-rank test. All tests were two-sided, with p < 0.05 considered statistically significant. 

## Results

Patient characteristics

The cohort included 41 patients: 15 in the radical surgery group, 18 in the conservative surgery group, and eight in the PAIR group. The participant selection process is illustrated in Figure 1.

**Figure 1:**
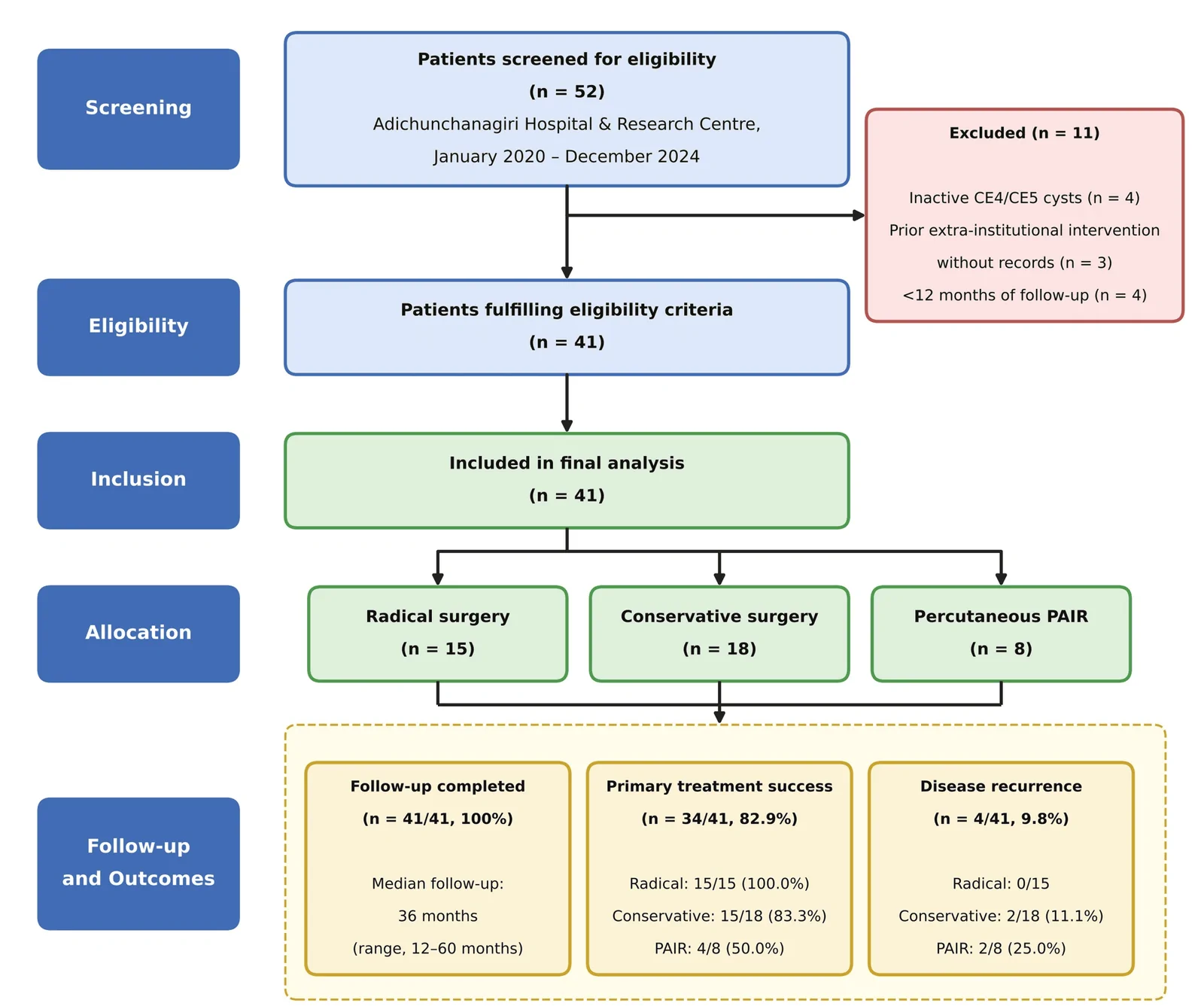
STROBE flow diagram of patient screening, eligibility, and treatment-group allocation in the retrospective hepatic cystic echinococcosis cohort Of 52 patients screened (January 2020 to December 2024), 11 were excluded (inactive CE4/CE5 cysts, n = 4; prior extra-institutional intervention without records, n = 3; <12 months of follow-up, n = 4). The 41 included patients were grouped by definitive intervention: radical surgery (n = 15), conservative surgery (n = 18), and percutaneous PAIR (n = 8). STROBE: Strengthening the Reporting of Observational Studies in Epidemiology; CE: cystic echinococcosis; PAIR: puncture, aspiration, injection, and re-aspiration; WHO-IWGE: World Health Organization Informal Working Group on Echinococcosis

Mean age was 53.2 ± 11.8 years, 24 patients (58.5%) were female, and 34 (82.9%) presented with abdominal pain. The measured demographic, clinical, and laboratory characteristics did not differ significantly among the three groups (Table 1).

**Table 1:** Baseline demographic, clinical, and laboratory characteristics by treatment group Continuous variables are reported as mean ± standard deviation, and categorical variables as number (percentage). Percentages are calculated using the treatment-group denominator. SD: standard deviation; IgG: immunoglobulin G; PAIR: puncture, aspiration, injection, and re-aspiration

Characteristic	Total (N = 41)	Radical surgery (n = 15)	Conservative surgery (n = 18)	PAIR (n = 8)	Statistic	p-value
Age, years, mean ± SD	53.2 ± 11.8	52.6 ± 12.1	54.1 ± 11.2	52.3 ± 13.1	F = 0.108	0.898
Female/male, n (%)	24/17 (58.5/41.5)	9/6 (60.0/40.0)	11/7 (61.1/38.9)	4/4 (50.0/50.0)	χ² = 0.309	0.857
Body mass index, kg/m², mean ± SD	24.8 ± 3.1	25.1 ± 3.4	24.4 ± 2.8	25.2 ± 3.2	F = 0.287	0.752
Abdominal pain, n (%)	34 (82.9)	12 (80.0)	15 (83.3)	7 (87.5)	χ² = 0.228	0.892
Palpable mass, n (%)	15 (36.6)	6 (40.0)	7 (38.9)	2 (25.0)	χ² = 0.612	0.736
Total bilirubin, mg/dL, mean ± SD	1.1 ± 0.4	1.0 ± 0.3	1.2 ± 0.5	1.1 ± 0.4	F = 0.814	0.450
Hydatid IgG positive, n (%)	33 (80.5)	12 (80.0)	15 (83.3)	6 (75.0)	χ² = 0.281	0.869

Imaging characteristics and WHO-IWGE stage

Mean cyst diameter was 9.6 ± 3.2 cm, and 26 patients (63.4%) had right-lobe involvement. Solitary cysts were present in 31 patients (75.6%) (Table 2). WHO-IWGE stage distribution differed among treatment groups (p < 0.001). CE3b accounted for 12/15 patients (80.0%) in the radical surgery group, 10/18 (55.6%) in the conservative surgery group, and 2/8 (25.0%) in the PAIR group. A preoperative computed tomography image is shown in Figure 2.

**Table 2:** Baseline imaging characteristics and WHO-IWGE stage by treatment group Continuous variables were compared using one-way ANOVA (F-statistic). Categorical stage distribution across groups was evaluated using the Freeman-Halton extension of Fisher's exact test (χ2-statistic equivalent reported). CE: cystic echinococcosis; PAIR: puncture, aspiration, injection, and re-aspiration; WHO-IWGE: World Health Organization Informal Working Group on Echinococcosis

Imaging Characteristic	Total (N=41)	Radical Surgery (n=15)	Conservative Surgery (n=18)	PAIR (n=8)	Statistic	p-value
Cyst diameter (cm), mean ± SD	9.6±3.2	10.2±3.5	9.5±3.0	8.8±2.8	F=0.548	0.582
Right-lobe involvement, n (%)	26 (63.4)	9 (60.0)	12 (66.7)	5 (62.5)	χ2=0.184	0.912
Solitary cyst, n (%)	31 (75.6)	11 (73.3)	14 (77.8)	6 (75.0)	χ2=0.089	0.956
WHO-IWGE stage, n (%)					χ2=18.241	<0.001
— CE1, active unilocular	6 (14.6)	0 (0.0)	2 (11.1)	4 (50.0)	—	—
— CE2, active multivesicular	11 (26.8)	3 (20.0)	6 (33.3)	2 (25.0)	—	—
— CE3b, daughter cysts in solid matrix	24 (58.5)	12 (80.0)	10 (55.6)	2 (25.0)	—	—

**Figure 2:**
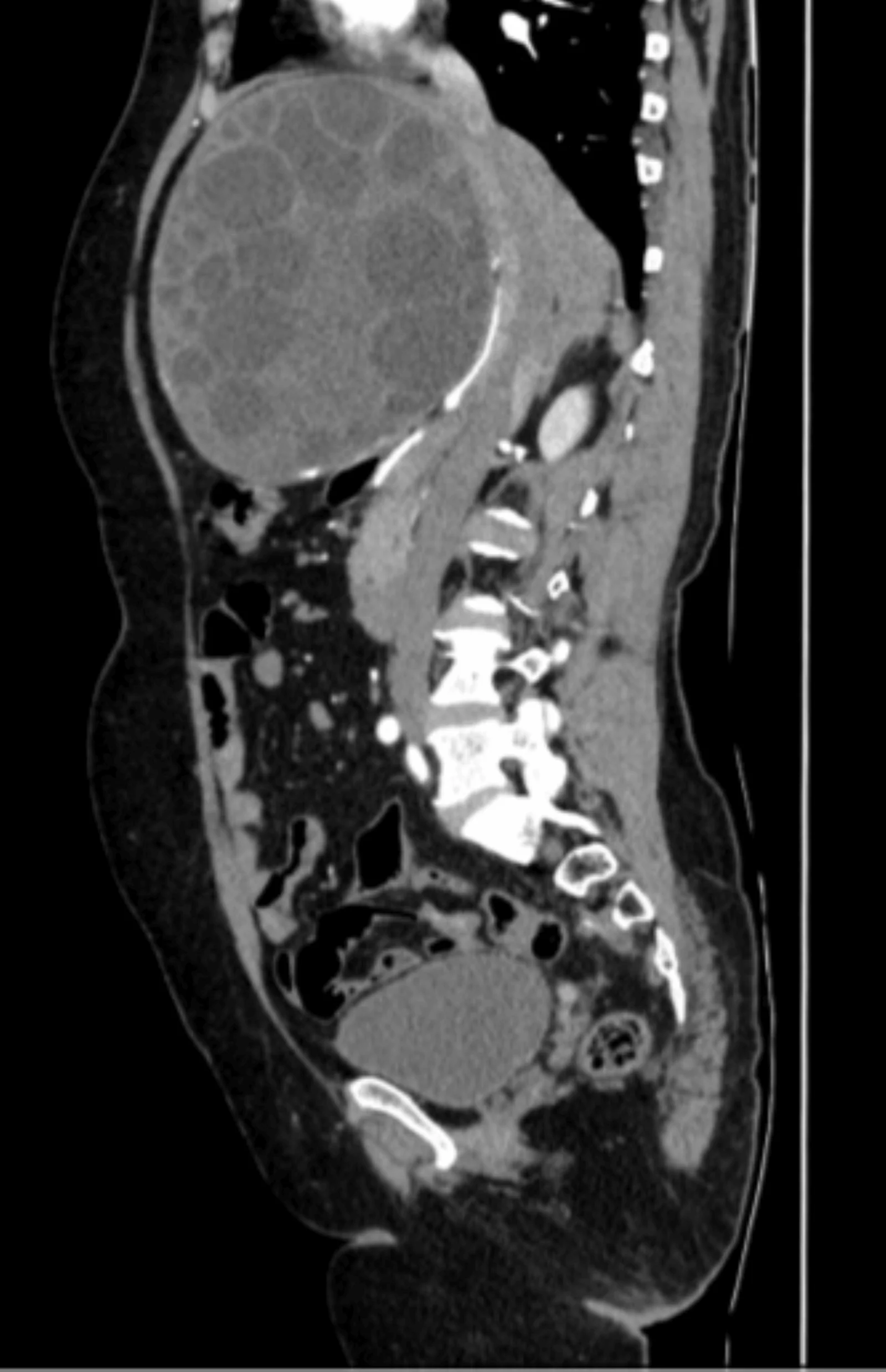
Preoperative computed tomography showing a large hepatic hydatid cyst containing multiple daughter cysts

Procedural characteristics

Mean procedure duration was 188.4 ± 36.2 minutes for radical surgery, 135.2 ± 26.5 minutes for conservative surgery, and 42.5 ± 12.0 minutes for PAIR. Mean estimated blood loss was 265.0 ± 82.1 mL, 158.0 ± 48.2 mL, and 12.5 ± 5.0 mL, respectively. The overall group comparisons were statistically significant for both measures (p < 0.001). Transfusion was recorded in 2/15 patients (13.3%) after radical surgery, 1/18 (5.6%) after conservative surgery, and none after PAIR (p = 0.412; Table 3). The laparoscopic appearance of a cyst with daughter cysts is shown in Figure 3. Operative and specimen photographs from a left lateral sectionectomy are shown in Figure 4, and the right hepatectomy specimen is shown in Figure 5.

**Table 3:** Procedural characteristics by treatment group Procedure duration and estimated blood loss were compared across groups using one-way ANOVA (F-value). Categorical proportions were evaluated using Fisher's exact test. Procedural and hospital-stay data for the PAIR group include/exclude the three patients converted to open surgery. PAIR: puncture, aspiration, injection, and re-aspiration

Procedural Measure	Radical Surgery (n=15)	Conservative Surgery (n=18)	PAIR (n=8)	Statistic	p-value
Procedure duration (min), mean ± SD	188.4±36.2	135.2±26.5	42.5±12.0	F = 67.5	<0.001
Estimated blood loss (mL), mean ± SD	265.0±82.1	158.0±48.2	12.5±5.0	F = 47.7	<0.001
Blood transfusion, n (%)	2 (13.3)	1 (5.6)	0 (0.0)	χ2=1.774	0.412

**Figure 3:**
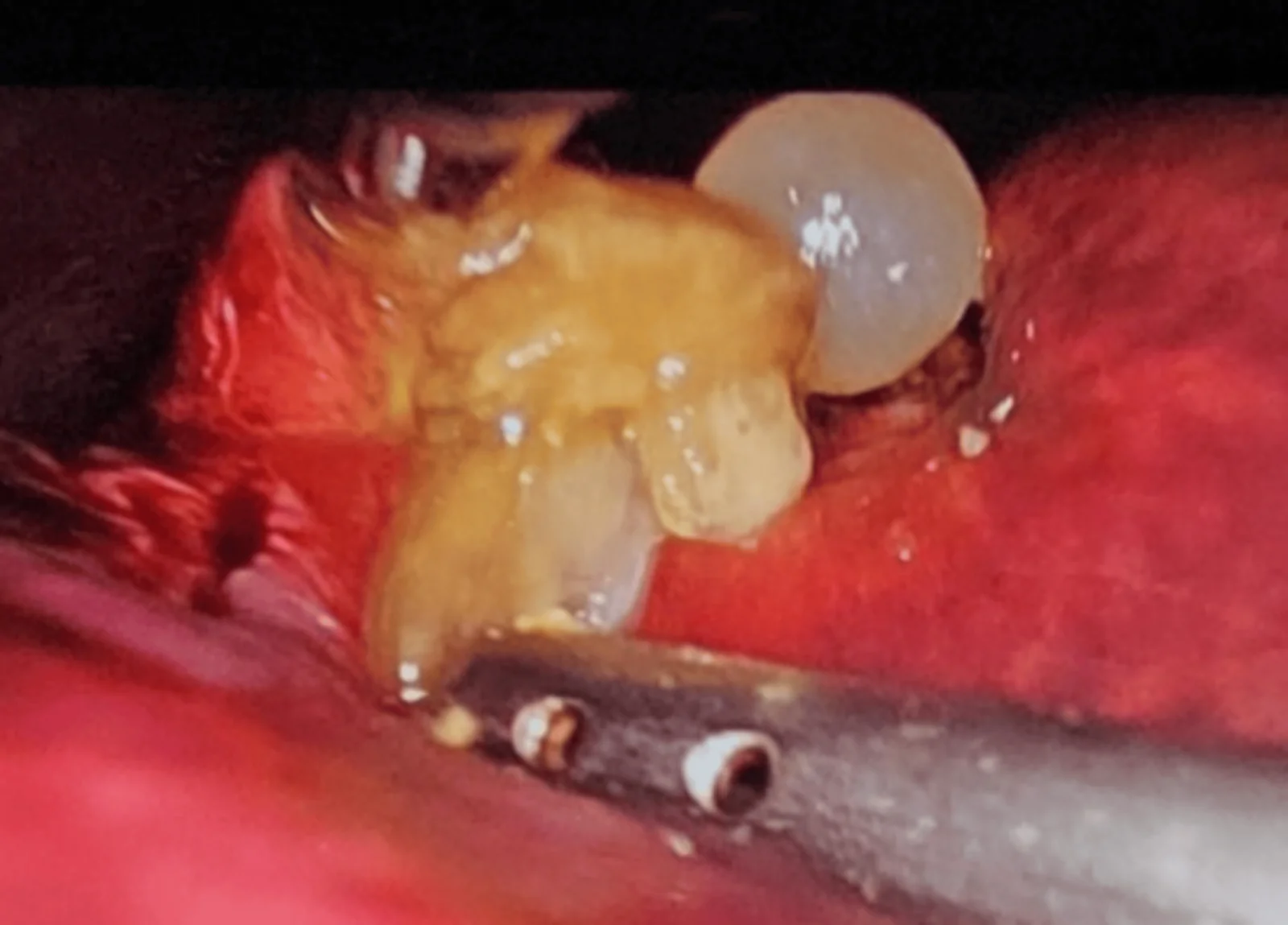
Intraoperative laparoscopic image showing a hepatic hydatid cyst and its daughter cysts

**Figure 4:**
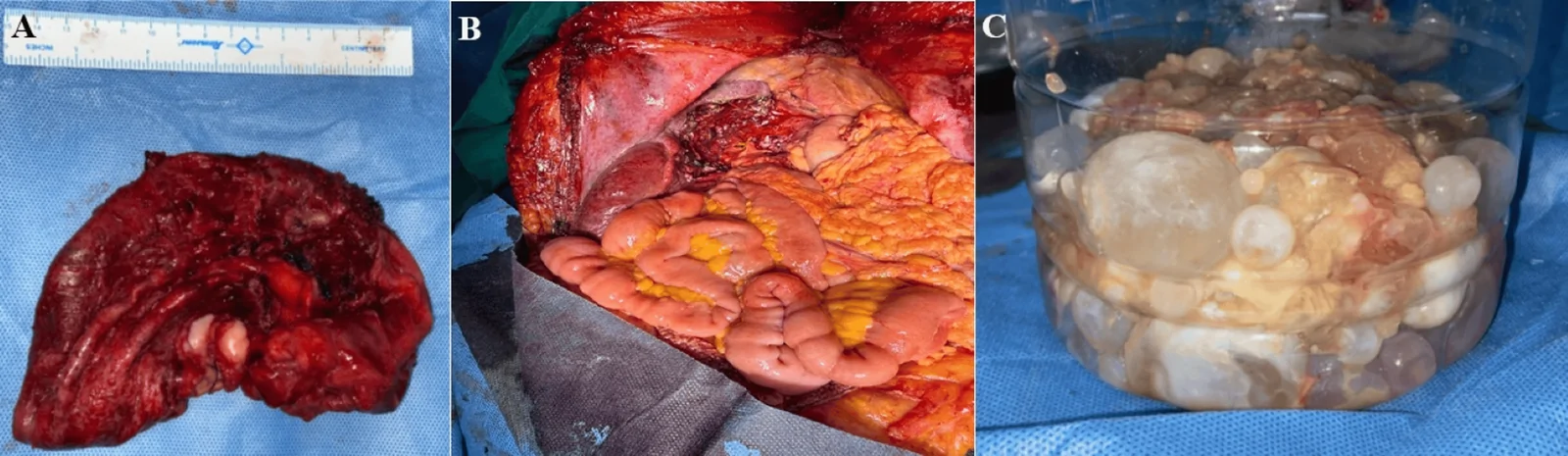
Operative and specimen photographs associated with left lateral sectionectomy A: resected liver specimen; B: intraoperative view of the operative field; C: evacuated cyst contents with daughter cysts.

**Figure 5:**
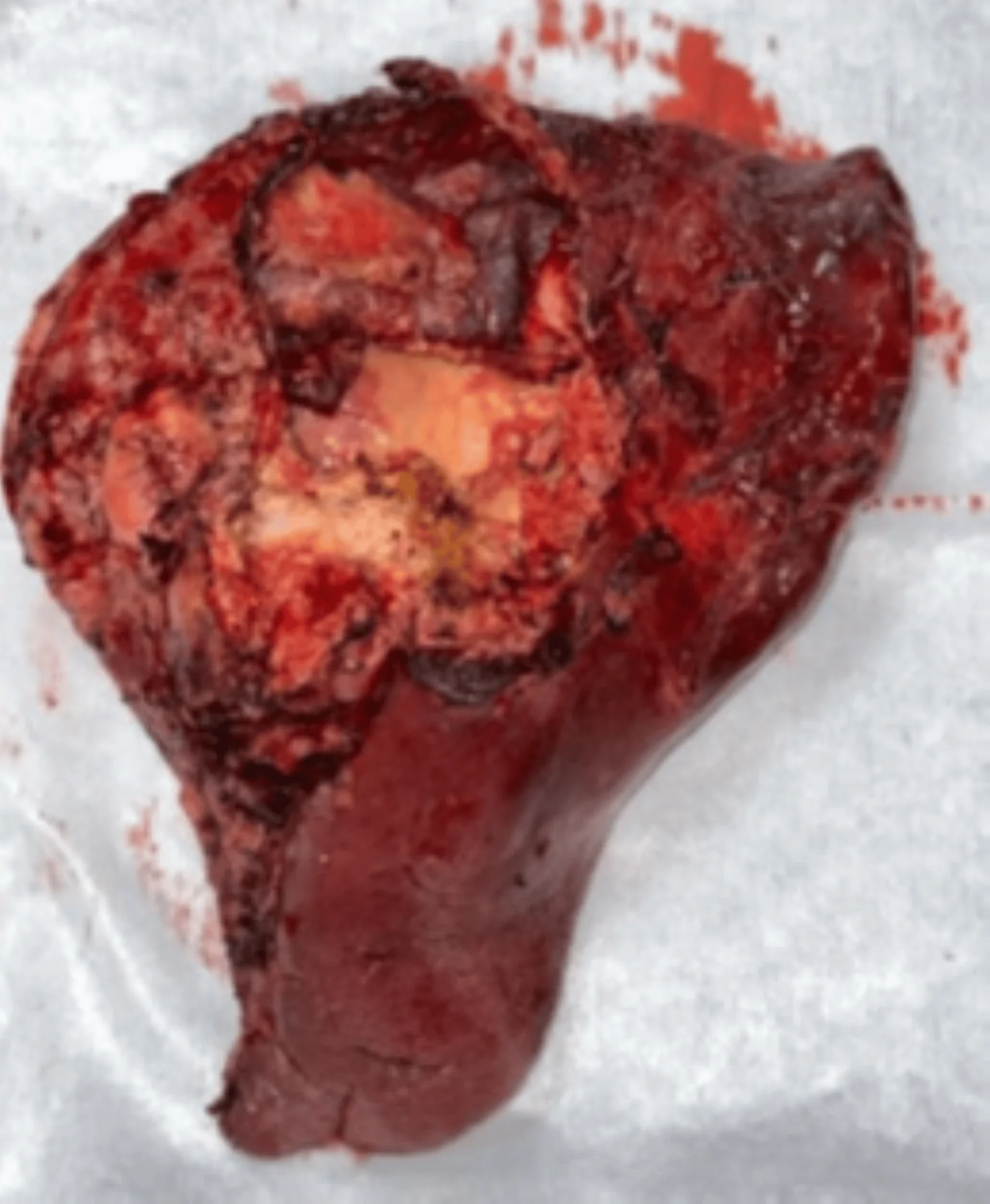
Resected right liver specimen following right hepatectomy for hepatic hydatid disease

Complications and hospital outcomes

Complications occurred in 13/41 patients (31.7%): 2/15 (13.3%) in the radical surgery group, 6/18 (33.3%) in the conservative surgery group, and 5/8 (62.5%) in the PAIR group (p = 0.038). Biliary leak or fistula was recorded in 0/15, 4/18 (22.2%), and 2/8 (25.0%), respectively (p = 0.124). Three patients in the PAIR group underwent conversion to surgery (37.5%), compared with none in the surgical groups (p = 0.003. Mean hospital stay was 6.1 ± 1.4 days after radical surgery, 9.8 ± 2.4 days after conservative surgery, and 3.5 ± 1.2 days after PAIR (p < 0.001; Table 4).

**Table 4:** Treatment-related complications and hospital outcomes by treatment group Group comparisons were performed using one-way ANOVA (F-value) for hospital stay length and Fisher's exact test (χ2-value) for complication rates. Procedural and hospital-stay data for the PAIR group include/exclude the three patients converted to open surgery. PAIR: puncture, aspiration, injection, and re-aspiration

Outcome	Total (N=41)	Radical Surgery (n=15)	Conservative Surgery (n=18)	PAIR (n=8)	Statistic	p-value
Overall complications, n (%)	13 (31.7)	2 (13.3)	6 (33.3)	5 (62.5)	χ2=5.86	0.053
— Grade I–II	7 (17.1)	2 (13.3)	2 (11.1)	3 (37.5)	χ2=2.891	0.236
— Grade IIIa–IIIb	6 (14.6)	0 (0.0)	4 (22.2)	2 (25.0)	χ2=3.204	0.201
Biliary leak or fistula, n (%)	6 (14.6)	0 (0.0)	4 (22.2)	2 (25.0)	χ² = 4.09	p = 0.129
Conversion to surgery, n (%)	3 (7.3)	0 (0.0)	0 (0.0)	3 (37.5)	χ² = 13.35	p = 0.001
Hospital stay (days), mean ± SD	7.2±3.1	6.1±1.4	9.8±2.4	3.5±1.2	F = 35.0	<0.001

Treatment success and recurrence

Median follow-up was 36 months (range, 12-60 months). Primary treatment success was observed in 15/15 patients (100.0%) in the radical surgery group, 15/18 (83.3%) in the conservative surgery group, and 4/8 (50.0%) in the PAIR group (p = 0.007). Recurrence was recorded in 0/15, 2/18 (11.1%), and 2/8 (25.0%), respectively. The crude comparison of recurrence counts had p = 0.116; the Kaplan-Meier log-rank comparison of recurrence-free survival had p = 0.048 (Table 5). All three conversions to surgery occurred in the PAIR group, and both PAIR recurrences occurred among the converted patients. Of the four PAIR patients who did not achieve treatment success, three required conversion. The reported success count means four PAIR patients did not meet the success definition, while three underwent conversion. 

**Table 5:** Treatment success and cyst recurrence by treatment group Primary treatment success is defined as complete radiological cyst collapse/obliteration without requiring secondary intervention. Follow-up was compared using the Kruskal–Wallis test (H-statistic). Recurrence-free survival was evaluated using the Kaplan- Meier log-rank test.

Outcome Parameter	Total (N=41)	Radical Surgery (n=15)	Conservative Surgery (n=18)	PAIR (n=8)	Statistic	p-value
Primary treatment success, n (%)	34 (82.9)	15 (100.0)	15 (83.3)	4 (50.0)	χ² = 9.22	p = 0.010
Cyst recurrence, n (%)	4 (9.8)	0 (0.0)	2 (11.1)	2 (25.0)	χ² = 3.77	p = 0.152
Recurrence-free survival	—	—	—	—	Log-rank χ2=6.071	0.048
Follow-up duration (months), median (IQR)	36 (24–48)	38 (26–50)	35 (22–46)	32 (18–44)	H=1.205	0.547

## Discussion

In this single-center retrospective cohort of 41 adults with active or transitional hepatic CE, observed treatment success was highest after radical surgery, followed by conservative surgery and PAIR. Overall complications and treatment conversion were also observed more frequently in the PAIR group, while mean hospital stay was shortest in that group. No biliary leaks were recorded after radical surgery, but the between-group comparison of biliary leak or fistula was not statistically significant. These findings describe outcomes in this cohort and should not be interpreted as evidence that one treatment caused better outcomes.

The treatment groups differed in WHO-IWGE stage distribution. CE3b accounted for 80.0% of the radical surgery group, 55.6% of the conservative surgery group, and 25.0% of the PAIR group, while CE1 was more frequent in the PAIR group. This imbalance reflects the clinical selection of treatment and limits direct comparison. Cyst stage, size, location, biliary communication, complications, and proximity to major vascular structures may all influence both treatment choice and outcome. Although CE3b was most frequent in the radical surgery group, the available data do not account for all anatomical and clinical factors that may have affected selection. The observed differences should therefore be considered unadjusted associations. Because the radical surgery group contained the highest proportion of CE3b cysts yet had the best observed outcomes, stage imbalance alone is unlikely to explain this pattern; selection by the treating surgeons according to cyst anatomy, location, and proximity to vascular and biliary structures is a more plausible confounder.

The results are consistent with some retrospective comparisons of radical and conservative surgery. Aydin and colleagues reported lower recurrence and morbidity after radical surgery than after conservative surgery in a retrospective cohort, with recurrence reported in 3.2% and 24.0% of patients, respectively [7]. A meta-analysis of comparative studies also found lower pooled rates of postoperative complications, biliary fistula, and recurrence with radical surgery, along with longer operation times; it found no significant differences in mortality or hospital stay [9]. However, the evidence base included studies with differing selection criteria, cyst characteristics, procedures, and follow-up periods. In contrast, a propensity score-matched study of 170 patients found recurrence in 4% of both the radical and conservative groups, with no significant differences in overall complications, reinterventions, or hospital stay [10]. These differing results support a cautious interpretation of the present cohort and underscore the difficulty of separating treatment effects from treatment selection in observational studies.

In the current cohort, radical procedures were associated with longer procedure duration and greater estimated blood loss than conservative surgery and PAIR, while hospital stay was longest after conservative surgery and shortest after PAIR. These are descriptive comparisons across procedures with different levels of invasiveness and different patient selection. The shorter stay following PAIR should be considered alongside the observed conversions and treatment failures. The longer stay after conservative surgery than after radical surgery (9.8 vs 6.1 days) may reflect biliary leak or fistula (4/18 in the conservative group vs none after radical surgery) and the management of the residual cavity and drains, but this could not be tested in this cohort. Gupta and colleagues reported that PAIR was abandoned in some patients when bile or pultaceous material was aspirated and that some unsuccessful PAIR procedures were followed by surgery [11]. This provides a relevant comparison for the procedural challenges observed in the present cohort. However, their study used a different cyst classification and treatment selection, so it does not establish the relative effectiveness of PAIR for the WHO-IWGE stages represented here.

Current WHO guidance likewise supports stage- and context-specific treatment rather than a single approach for all active cysts. It states that surgery is usually the treatment of choice for complicated hepatic cysts and provides separate recommendations for uncomplicated disease according to cyst stage, size, and available expertise [5]. The present study includes a mixed-stage cohort and cannot establish the best treatment for an individual stage. In particular, only two patients with CE3b received PAIR; outcomes in that subgroup are too few to support a general conclusion about PAIR for CE3b cysts. Because both PAIR recurrences occurred in converted patients, the recurrence attributed to PAIR under intention-to-treat reflects the combined PAIR-then-surgery pathway.

The study has several strengths. It includes consecutive patients treated over a five-year period at a tertiary-care center, compares three treatment strategies used in clinical practice, and reports perioperative outcomes alongside recurrence during a median follow-up of 36 months. Use of WHO-IWGE staging and reporting of conversion, biliary events, hospital stay, and recurrence provides clinically relevant detail.

The findings should also be interpreted in light of important limitations. The retrospective, single-center design is vulnerable to selection bias, incomplete records, and unmeasured or unrecorded confounding. The treatment groups were small and unequal, and stage distribution differed significantly. The three strategies have different indications and are not interchangeable alternatives for the same patient, so with 41 patients the three-way comparison has limited validity beyond that imposed by sample size alone. Treatment allocation was not protocol-driven, and the cyst stage was taken from the original imaging reports without independent re-review. Operative technique details (residual cavity management, intraoperative scolicidal use, and drain policy), PAIR procedural details (catheter use and pre-injection biliary assessment), and the criteria and decision-makers for treatment allocation were not uniformly documented in the retrospective records. Potentially important confounders were also not captured, including complicated presentation (rupture, infection, preoperative biliary communication), segmental location and relation to major vessels and bile ducts, comorbidity, and operative approach (laparoscopic or open). PAIR was used in some patients with CE2 or CE3b cysts (four of eight), stages for which it is less established, so its observed success rate (50%) is likely to reflect patient selection as well as the technique itself. Treatment success is a composite that includes absence of recurrence and was analyzed as a proportion irrespective of follow-up duration; follow-up was documented for at least 12 months in all patients. In addition, intention-to-treat analysis attributes the three conversions to open surgery, and both PAIR recurrences, which occurred in converted patients, to the PAIR group; the procedural and hospital-stay data for the PAIR group should be read with this in mind. Only four recurrences were reported, making recurrence estimates and the log-rank comparison imprecise; the 12-60-month follow-up range also means patients had differing opportunities for recurrence to be detected. The study was not randomized and does not establish treatment superiority. In addition, the reported crude recurrence comparison (p = 0.116) and Kaplan-Meier log-rank result (p = 0.048) are based on different analyses and should be interpreted alongside event timing, censoring, confidence intervals, and numbers at risk. Generalizability may be limited to centers with similar patient populations and surgical and interventional expertise.

Overall, this cohort provides real-world outcome data across radical surgery, conservative surgery, and PAIR, but the observed differences are hypothesis-generating. Larger, preferably multicenter, studies with clear stage-specific eligibility, standardized outcome definitions, comparable follow-up, and adjustment for treatment-selection factors are needed to estimate comparative outcomes more reliably.

## Conclusions

In this retrospective cohort, outcomes varied across radical surgery, conservative surgery, and PAIR. Radical surgery showed the most favorable observed outcome pattern, but differences in cyst stage and clinical selection prevent this study from establishing that it is superior.

Treatment should be individualized according to cyst stage, anatomy, complications, and available expertise. Larger multicenter studies with standardized outcome definitions and longer, comparable follow-up are needed to clarify the relative benefits and risks of these approaches.
